# Land Scurvy

**Published:** 1861-07

**Authors:** 


Land Scurvy.—This form of disease will be found among troops who suffer from the want of fresh meat, good flour or bread, and vegetable food in its natural state. The blood becomes depraved and the system debilitated, with a tendency to hemorrhage and a low form of inflammation in various parts of the body.

But little medical treatment is required in this disease. Great cleanliness, diet of sour krout, spinage, celery, garlic, onions, carrots, and potatoes. According to experiments made in the Milbank Penitentiary, by the use of this latter vegetable, the physician in charge succeeded in banishing the disease from it. One of the best applications to the gums is the nitrate of silver or sulphate of copper. A gentle laxative should be given to keep the bowels open.

In the recent report of the sickness and mortality among the United States troops, it is stated, on the authority of Assistant-Surgeon E. W. Johns, that "even vegetable matter, restricted to one form, may not prevent scurvy, as was the case among the lime groves at Fort Dallas, Florida, where the parade was covered with lemon, lime, and orange trees. With reference to this case, however, as well as his recollection now serves him, the troops at Fort Dallas were without fresh beef, and the flour was bad.

---

* Vattek, by William Beckford, of England.

Potash, he also observes, and citric acid, in his experience of several years in Texas, had not the slightest value. He believes that the greatest developing cause, in the case of the soldier, is guard-duty at night.

It was found by Dr. John J. Gaensten, while on duty at Camp Cooper, on the Clear Fork of the Brazos, that "in the absence of such anti-scorbutics as the army is usually supplied with, a remedy, free of cost, was found close by in abundance. The young and tender shoots of phytolacca, and of the various species of rumex were recommended, and when prepared, were freely eaten; one patient, unable either to walk or to discern objects, on the free use of the articles mentioned, was returned for duty in four weeks."—*Med. and Surg. Rep.*

